# Apoptotic Effect of 1800 MHz Electromagnetic Radiation on NIH/3T3 Cells

**DOI:** 10.3390/ijerph17030819

**Published:** 2020-01-28

**Authors:** Dan-Yang Li, Jing-Dong Song, Zhao-Yuan Liang, Kiana Oskouei, Xiang-Qian Xiao, Wen-Zhe Hou, Jin-Tao Li, Yi-Shu Yang, Ming-Lian Wang, Manuel Murbach

**Affiliations:** 1 College of Life Science and Bioengineering, Beijing University of Technology, Beijing 100124, China; dayangde@emails.bjut.edu.cn (D.-Y.L.); zyliang@emails.bjut.edu.cn (Z.-Y.L.); kianousk@hotmail.com (K.O.); xiaoxq@bjut.edu.cn (X.-Q.X.); ljt2008@bjut.edu.cn (J.-T.L.); yishu-y@bjut.edu.cn (Y.-S.Y.); 2 State Key Laboratory of Infectious Disease Prevention and Control, National Institute for Viral Disease Control and Prevention, Chinese Center for Disease Control and Prevention, Beijing 100052, China; songjd@ivdc.chinacdc.cn (J.-D.S.); houwz@ivdc.chinacdc.cn (W.-Z.H.); 3 Beijing International Science and Technology Cooperation Base of Antivirus Drug, Beijing University of Technology, Beijing 100124, China; 4 IT’IS Foundation, Zeughausstrasse 43, 8004 Zurich, Switzerland

**Keywords:** electromagnetic radiation, cell apoptosis, mitochondria, p53

## Abstract

To investigate the effect of 1800 MHz electromagnetic radiation (EMR) on apoptosis, we exposed NIH/3T3 cells at 1800 MHz with a specific absorption rate (SAR) of 2 W/kg intermittently for 12, 24, 36, and 48 h. After exposure, Cell Counting Kit-8 (CCK-8) and flow cytometry were used to detect cell viability and apoptosis; the expression of p53, a molecule with the key role in apoptosis, was measured by real-time qPCR, western blot, and immunofluorescence; and images of the structure of the mitochondria, directly reflecting apoptosis, were captured by electron microscopy. The results showed that the viability of cells in the 12, 36, and 48 h exposure groups significantly decreased compared with the sham groups; after 48 h of exposure, the percentage of late apoptotic cells in the exposure group was significantly higher. Real-time qPCR results showed that p53 mRNA in the 48 h exposure group was 1.4-fold of that in the sham group; significant differences of p53 protein fluorescence expression were observed between the exposure groups and the sham groups after 24 h and 48 h. The mitochondrial swelling and vesicular morphology were found in the electron microscopy images after 48 h exposure. These findings demonstrated 1800 MHz, SAR 2 W/kg EMR for 48 h may cause apoptosis in NIH/3T3 cells and that this apoptosis might be attributed to mitochondrial damage and upregulation of p53 expression.

## 1. Introduction

Nowadays, people are more frequently exposed to all kinds of electromagnetic radiation (EMR), mainly emitted from mobile phones, which are suspected hazards to human health, classified as possibly carcinogenic to humans (Group 2B) by the International Agency for Research on Cancer (IARC) [1]. However, whether EMR affects health remains controversial. Some epidemiological studies have demonstrated that EMR may cause brain tumors [2,3], breast cancer [4], adrenal gland and heart tumors [5], etc. In addition, EMR may have adverse effects on the central nerve system [6] and the reproductive system [7,8] and give rise to stress and anxiety in behavior [9]. However, some other reports have given negative results, e.g., radiofrequency electromagnetic field (RF-EMF) could not give rise to cell apoptosis [10], global system communication (GSM) exposure did not significantly increase the apoptosis rate of neurons [11], mobile phone radiation did not induce pro-apoptosis effects in spermatozoa [12], 900 MHz electromagnetic fields (EMF) did not affect survival rates in female mice [13], and RF exposure did not affect the depression-like behavior, spatial memory, and brain histology in adolescent male mice [14]. The potential biological effects of EMR have not been proved and current research data are insufficient to provide strong evidence of the possible health risks. The above findings indicate that there is a need for more scientific research support on the bioeffects of EMR.

Among the mobile phone wave bands, ranging from 300 MHz to 3 G, 1800 MHz is often used in China and Eastern European countries. In order to establish communication with the base station, the mobile phone as a radiation source emits electromagnetic waves to form an RF-EMF around it. The intensity of the radiation power of a mobile phone is generally defined as “Specific Absorption Rate” (SAR), to indicate the average ratio of radiant energy absorbed by the soft tissue of the head, in units of W/kg or mW/g. The maximum SAR limit for a mobile phone in accordance with the International Commission on Non-Ionizing Radiation Protection (ICNIRP) guidelines is 2 W/kg [15], averaged over 10 g of tissue and over any 6 min period. On a more local level (<1 g tissue, e.g., for some parts of the ear), cells can be exposed to significantly higher SAR values.

It has been established that cell apoptosis increases after exposure to EMR [16,17], accompanied by upregulation of a series of apoptosis-related genes such as p53, bax, and caspase-3 [18,19]. p53 is a tumor suppressor gene and it plays an important role in DNA damage, the cell cycle, and apoptosis [20,21]. Moreover, it is well known that mitochondrial damage is a key reason for cell apoptosis. At present, the conventional method for detecting mitochondria is to stain with fluorescence to roughly estimate mitochondrial membrane potential, but this method is low in sensitivity and not easy to recognize. The most accurate and intuitive method is to examine the mitochondria ultrastructure by electron microscopy [22]. Normal mitochondria are made of two membranes. The outer membrane covers the organelle, and the inner membrane, which houses the electron transport chain (ETC), folds over many times to create cristae. When damaged, mitochondrial swelling and vesicular structure occur [22]. In this study, we investigated apoptosis, the expression of p53, and mitochondria in NIH/3T3 cells exposed to 1800 MHz, SAR 2 W/kg EMR.

## 2. Materials and Methods

### 2.1. Cell Culture

Mouse embryonic fibroblast NIH/3T3 cells, purchased from the National Infrastructure of Cell Line Resource, were cultured in complete medium, composed of Dulbecco’s Modified Eagle Medium (Gibco, Grand Island, NY, USA), 10% fetal bovine serum (Gibco), 100 U/mL penicillin, and 100 μg/mL Streptomycin, at 37 °C with 5% CO_2_ in the humidified cell incubator (Thermo Scientific, Waltham, MA, USA) and passaged every 3–4 days.

### 2.2. Exposure System

The sXc1800 system (IT’IS Foundation, Zurich, Switzerland) was used to expose cells to EMR. It consists of an exposure setup, RF signal generator, RF amplifier, data acquisition, function generator, ventilation, computer, monitor, CO_2_ incubator, RF detectors, and temperature sensors. The CO_2_ incubator contains 2 waveguide chambers with equal temperature, humidity, and CO_2_ concentration. Six Petri dishes with Ф 35 mm can be inserted into each of the 2 waveguide chambers, one for EMR exposure and the other for sham exposure. The whole system has been fully characterized [23]. The numerical dosimetry for the field within the Petri dishes was verified using *E*-field. Cell monolayers with an entire cultivation area were exposed to a uniform SAR distribution. The environmental parameters (such as temperature and SAR value) were continuously monitored. We investigated the SAR level of 2 W/kg, at which any thermal effects can be excluded due to an assessed temperature increase of below 0.1 °C. Approximately 2 × 10^4^ cells were placed in each Ф 35 mm petri dish. After 24 h, cells were exposed to the 1800 MHz EMR for different periods of time (12, 24, 36, 48 h). The cells were intermittently (5 min on/10 min off) exposed to the EMR.

### 2.3. Cell Viability Assay

Cell Counting Kit-8 (CCK-8, Dojindo Laboratories, Beijing, China) was used to examine cell viability after EMR exposure. The cells were digested with 0.25% trypsin, and the cell suspensions (100 μL/well, 1 × 10^3^ cells per well) were seeded in a 96-well plate. After the cells adhered, 10 μL of CCK-8 solution was added to each well and the plate was incubated at 37 °C for 1 h. The viability of cells was determined by the absorbance measured at 450 nm using a microplate reader (BIO-RAD, Hercules, CA, USA).

### 2.4. Cell Apoptosis Assay

Apoptosis in NIH/3T3 cells was detected using an Annexin V-FITC assay kit (Dojindo Laboratories). After EMR treatment, the cells were digested with 0.25% trypsin, washed twice with phosphate buffer saline (PBS), then had 1 × Annexin V Binding Solution added to make a final cell concentration of 1 × 10^6^ cells/mL. A 100 μL sample of each cell suspension was stained with 5 μL Annexin V-FITC and 5 μL PI solution at room temperature for 15 min. After that, 400 μL of Annexin V Binding Solution was added to the cell suspensions and the results were analyzed by a flow cytometer (Becton, Dickinson and Company, Franklin Lakes, NJ, USA).

### 2.5. Real-Time qPCR to Detect p53 Expression

Total RNA was extracted using the standard TRIzol protocol to determine the RNA concentration and purity. RNA was converted into cDNA according to the instructions of the reverse transcription kit (TAKARA, Dalian, China). cDNA was amplified and quantified using the qPCR kit (TAKARA). p53 forward primer: 5′-TACAAGAAGTCACAGCACAT-3′, reverse primer: 5′-CCAGATACTCGGGATACAAAT-3′. GAPDH forward primer: 5′-TGGCCTTCCGTGTTCCTAC-3′, reverse primer: 5′-GAGTTGCTGTTGAAGTCGCA-3′. All qPCR reactions were performed using the ViiA7 real-time PCR system (Applied Biosystems, Foster City, CA, USA) with the following thermal cycle conditions: 95 °C for 10 min; 39 cycles of 95 °C for 15 s then 60 °C for 1 min; followed by a melt curve from 60 °C to 95 °C with an increment of 1.6 °C per cycle.

### 2.6. Western Blot to Detect p53 Protein Expression

After EMR exposure, NIH/3T3 cells were washed twice with cold PBS, then gently scraped with a cell scraper and lysed on ice with RIPA lysis buffer (Beyotime Biotechnology, Shanghai, China) containing phenylmethylsulfonyl fluoride (PMSF) for 30 min. The protein lysates were centrifuged at 4 °C 12,000× *g* for 30 min to separate the supernatant. The protein concentration of each was evaluated using a BCA protein assay kit (Solarbio life science, Beijing, China). Equivalent proteins (20 μg each lane) were subjected to SDS-Polyacrylamide Gel Electrophoresis (SDS-PAGE) on 10% gel and transferred to PVDF membranes. After blocking with 5% skim milk at room temperature for 2 h, the membranes were blotted with p53 primary antibody (Proteintech Group, Wuhan, China, 1:1000) and GAPDH primary antibody (Bioworld Technology, Nanjing, China, 1:10,000) overnight at 4 °C. After that, membranes were incubated with corresponding secondary antibodies at a 1:10,000 dilution. Finally, the membranes were scanned on the Odyssey infrared fluorescence imaging system (LI-COR, Lincoln, Nebraska, USA). The intensity of the western blot signals was quantitated using ImageJ software, version 1.41o (National Institutes of Health, Bethesda, MD, USA).

### 2.7. Confocal Microscopy to Analyze p53 Expression

After EMR exposure, pre-warmed mitochondrial dye MitoTracker Red CMXRos (Invitrogen Carlsbad, CA, USA) stained the NIH/3T3 cells at 37 °C for 45 min. Cells were fixed with 4% paraformaldehyde at 37 °C for 15 min, washed three times with PBS, and permeabilized with Triton X-100 at room temperature for 10 min. After washing three times with PBS, sheep serum was used for blocking at room temperature for 30 min, then p53 primary antibody (Proteintech Group, Wuhan, China, 1:100) was added and the cells incubated at 4 °C overnight. The cells were again washed three times with PBS for 5 min each time. The cells were incubated with the secondary antibody (EarthOx Life Sciences, Beijing, China, 1:100) for 1 h in the dark and washed 3 times with PBS for 5 min each time. The nucleus was stained for 30 min using Hoechst stain and washed three times with PBS. The results were analyzed by a laser confocal microscope (Zeiss LSM880, Jena, Germany).

### 2.8. Analysis of Mitochondrial Structure by Electron Microscope

The irradiated NIH/3T3 cells were fixed with 2% glutaraldehyde for 2–4 h and washed three times with 0.1 M sodium cacodylate buffer (pH 7.4) for 10 min. The fixed cells were then incubated with 1% osmium tetroxide at room temperature for 2 h. After washing with distilled water three times, fixed cells were dehydrated in ethanol series of 50%, 70%, 90%, 100%, 100%, and 100% successively for 10 min each. The cells were infiltrated in 50% ethanol, 50% 812 embedding agent for 1 h; 25% ethanol, 75% 812 embedding agent for 1 h; then 100% 812 embedding agent overnight. The samples were polymerized at 60 °C for 48 h, after that the samples were sectioned (approximately 80 nm). Thin sections were collected and pre-stained with 2% uranyl acetate and lead citrate each for 10 min before examination by an electron microscope (FEI tecnai20, Hillsboro, Oregon, USA).

### 2.9. Statistical Analysis

The statistical software SPSS 24.0 (SPSS Inc., Chicago, IL, USA) was used to perform the statistical analyses. All of the experiments were conducted at least in triplicate. All data were presented as the mean ± SD of each group. Statistical analyses were performed with ANOVA and Student’s *t*-test. Differences at *p* < 0.05 were considered statistically significant.

## 3. Results

### 3.1. NIH/3T3 Cell Viability and Apoptosis

**Figure 1:**
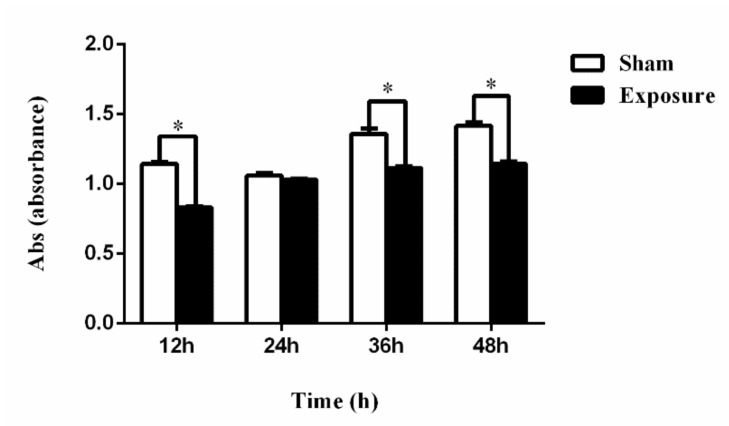
The 1800 MHz EMR could significantly inhibit NIH/3T3 cell viability at interval hours (12, 36, 48 h). Cell viability was determined using a Cell Counting Kit-8 (CCK-8) assay. Data are represented as mean ± SD (*n* = 3). * *p* < 0.05 compared with the sham groups using two-way ANOVA.

**Figure 2:**
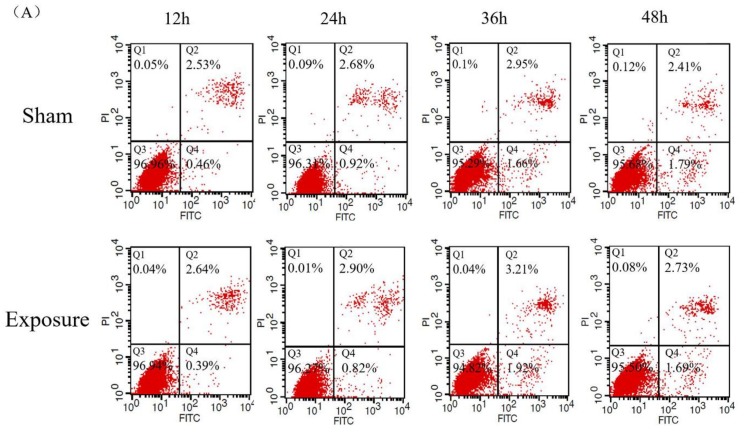

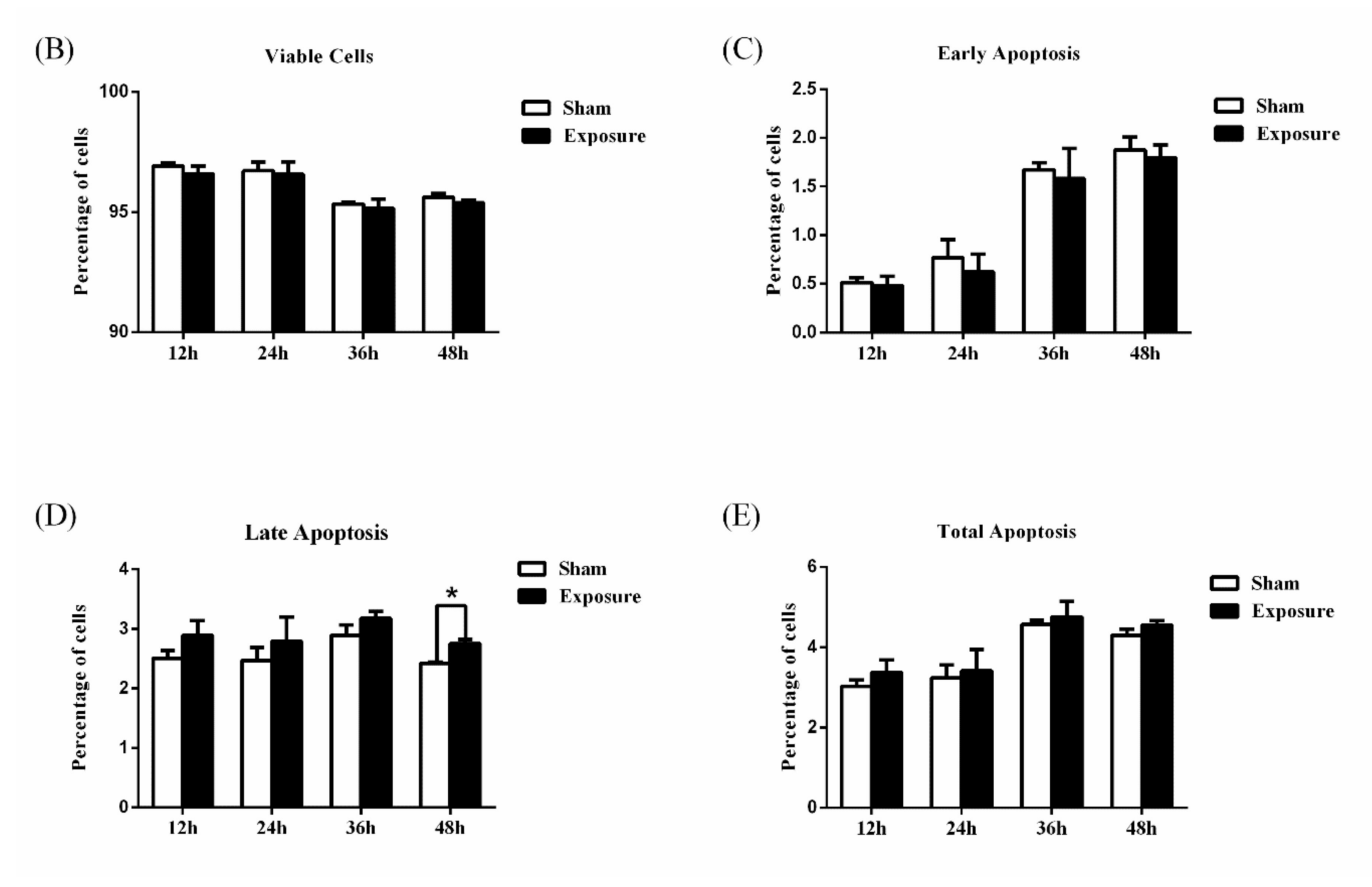
The 1800 MHz EMR may induce apoptosis in NIH/3T3 cells, especially late apoptosis, after 48 h. Annexin V-FITC staining and flow cytometry analyses were used to detect cell apoptosis. (**A**) Flow cytometry results. Q1: necrosis; Q2: late apoptosis; Q3: viable cells; Q4: early apoptosis. (**B**) The percentage of viable cells in each exposure group and sham group. (**C**) Early apoptotic rate. (**D**) Late apoptotic rate. (**E**) Total apoptotic rate. Data are expressed as mean ± SD (*n* = 3). * *p* < 0.05 compared with the sham group using two-way ANOVA.

Results of cell viability by 1800 MHz EMR are presented in Figure 1. We found that cell viability had decreased in the exposure groups compared with the sham groups after irradiation. In the groups exposed to EMR for 12, 36, and 48 h, cell activity showed a significant decline (*p* < 0.05). The reason for why the data for the 24 h groups presented a different trend (no significant difference) to the other periods (12, 36, and 48 h) is that the trend of cell viability was consistent with what we have shown in our other article on reactive oxygen species (ROS) [24]. The results of ROS fluctuated in different periods, not a stable trend. This is due to the neutralization of the effects of damage, coupled with cellular repair mechanisms. In addition, the sensitivity of cell viability is related to cell growth status in different cell cycles. We speculated that the 24 h cells were in a period of the cell cycle during which they were not sensitive to EMR, so cell viability might not have been easily affected. Cell apoptosis data are presented in Figure 2. The results showed that the percentage of late apoptotic cells and the total percentage of apoptotic cells in the exposure groups were higher than those in the sham groups (Figure 2D,E); the percentages of viable cells in the exposure groups were lower than those in the sham groups (Figure 2B), which was consistent with the experimental results of CCK-8. It is worth noting that there was a significant difference in late apoptotic cell percentage between the exposure group and the sham group after 48 h (*p* < 0.05). No significant difference was found in the other groups.

### 3.2. p53 Expression Analysis

**Figure 3:**
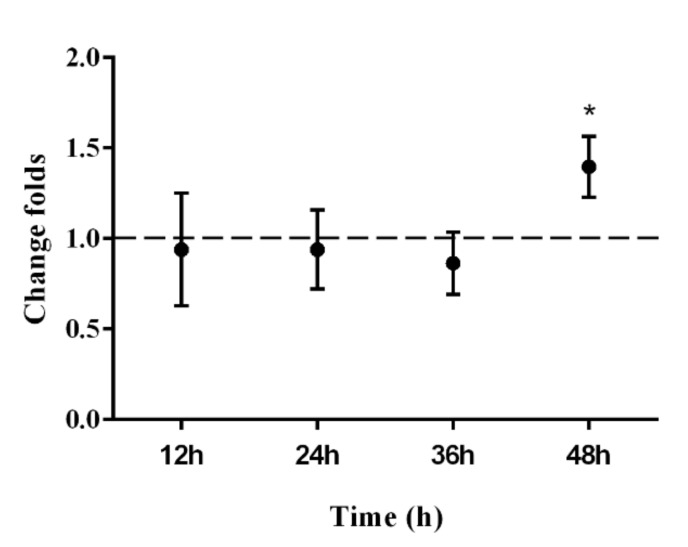
p53 mRNA expression increased significantly after exposure to 1800 MHz EMR for 48 h. Real-time qPCR was used to detect changes of p53 mRNA. Data are expressed as mean ± SD (*n* = 3). * *p* < 0.05 compared with the sham group using one-way ANOVA.

**Figure 4:**
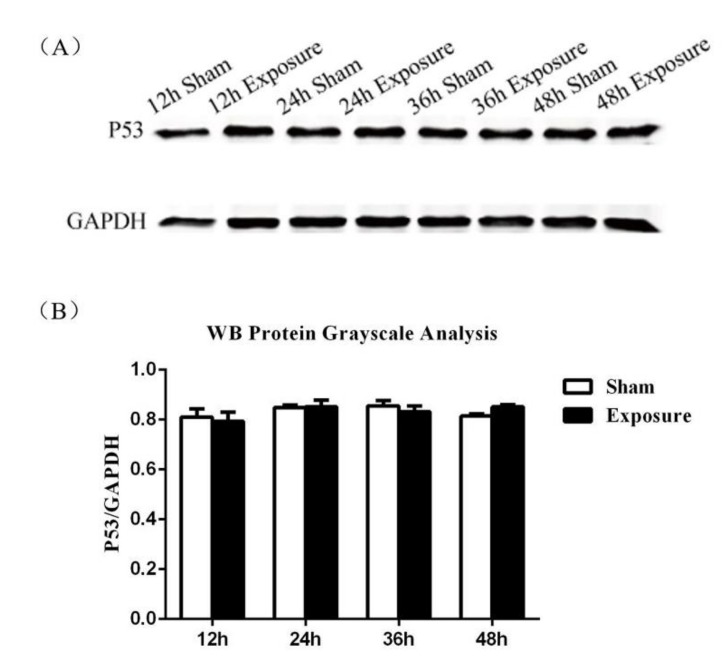
The expression of p53 protein increased after treatment with 1800 MHz EMR for 48 h. Western blot was used to detect p53 and GAPDH protein. (**A**) Western blot analysis. GAPDH was used as an internal control to monitor for equal loading. (**B**) The protein grayscale software analysis results. Data are expressed as mean ± SD (*n* = 3), compared with the sham groups using two-way ANOVA. No significant difference was observed between the sham and exposure groups (*p* > 0.05). *p*-values for 12 h, 24 h, 36 h, and 48 h were, respectively, 0.8636, 0.9997, 0.6382, and 0.2592.

**Figure 5:**
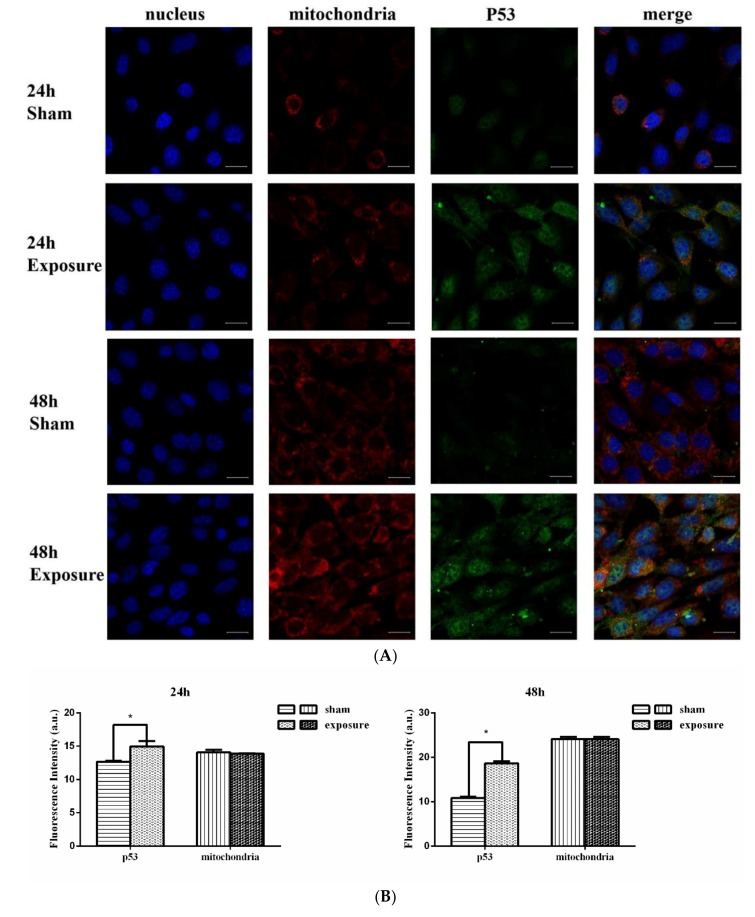
Immunofluorescence and confocal microscopy were used to analyze p53 protein expression. p53 protein expression increased significantly both in 24 h and 48 h at 1800 MHz EMR. The number of intact mitochondria decreased slightly after 24 h and 48 h. (**A**) The nucleus, mitochondria, and p53 protein were stained with Hoechst, MitoTracker Red CMXRos, and Dylight 488, respectively. (**B**) Quantitative analysis of p53 protein expression and intact mitochondria in the NIH/3T3 cells after exposure. Data are expressed as mean ± SD (n = 3; * *p* < 0.05 vs. sham groups) using *t*-test. Scale bar: 20 μm.

Data of real-time qPCR are presented in Figure 3. After 12, 24, and 36 h of EMR there was no significant difference in the expression of p53 at the mRNA level. However, after 48 h irradiation, p53 mRNA expression had increased significantly by 1.4-fold compared with the sham group. The results of the western blot analysis are shown in Figure 4. ImageJ software analysis, shown in Figure 4, showed there was no significant difference, *p*-values for 12 h, 24 h, 36 h, and 48 h groups were 0.8636, 0.9997, 0.6382, and 0.2592, respectively. The lowest *p*-value (*p* = 0.2592) was for the 48 h group, which is the closest to the confidence limit. It can be seen from the results of immunofluorescence (Figure 5) that p53 is distributed both inside and outside the nucleus. Fluorescence quantification was analyzed by ImageJ software, which showed that p53 protein expression in the exposure groups was enhanced significantly after 24 h and 48 h compared with the corresponding sham groups. Combining the three results for p53, we consider that p53 expression might increase in the exposure group after 48 h of EMR.

### 3.3. Mitochondria Imaging

**Figure 6:**
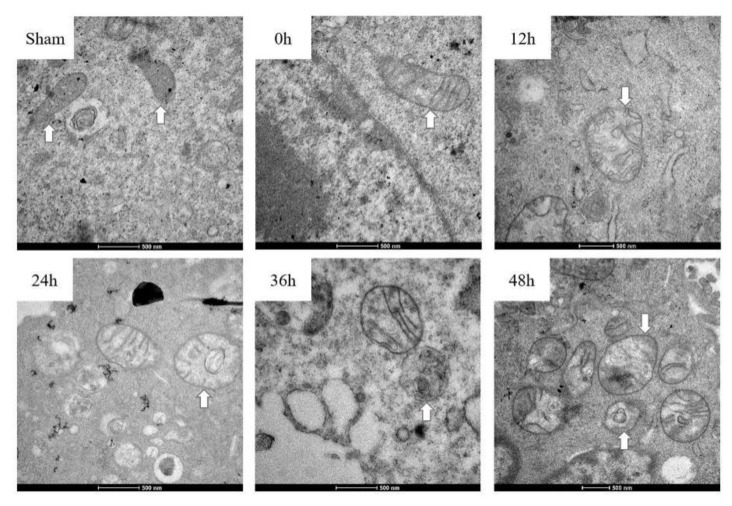
EMR at 1800 MHz may cause damage to mitochondria in NIH/3T3 cells. Electron microscopy was used to detect mitochondrial structure.

As presented in Figure 5, mitochondrial fluorescence intensity was slightly lower in the exposure groups. This represents a slight decrease in the number of normal mitochondria (with high mitochondrial membrane potential) in the exposure groups. The results of the electron microscopy of mitochondria are presented in Figure 6. The sham groups and 0 h group cells showed normal mitochondrial structure, while NIH/3T3 cells treated by EMR appeared to be swollen and had vesicular morphology. The longer the irradiation time, the more frequent the appearance of altered mitochondria morphology (e.g., vesicular morphology and swelling). This means that EMR may cause damage to the mitochondrial structure.

## 4. Discussion

In this study, no obvious difference in cell apoptosis rate was found between the exposure groups and the sham groups after 12, 24, and 36 h of irradiation, while the percentage of late apoptotic cells in the 48 h exposure group was significantly higher. Meanwhile, CCK-8 results showed that cell viability significantly declined after 12, 36, and 48 h EMR exposure. Similar to our findings, Liu et al. found that 1950 MHz EMR caused the percentage of apoptotic cells to significantly increase after 48 h [25]; our previous research results showed that 1800 MHz EMR for 1, 4, and 8 h caused a significant increase in the percentage of late apoptotic cells [24]; Lin et al. found that 1950 MHz, SAR 3 W/kg EMR for 24 h can cause adverse effects on TM3 cell proliferation [26]; Bilal et al. indicated that mobile phone radiation had an obvious apoptotic effect on MCF-7 cells [27]; Kesari et al. claimed that 3G cell phone network exposure caused cell apoptosis in rat brain [28]; and Buttiglione et al. proved that cell viability significantly decreased after 24 h of RF-EMF exposure [29]. In order to qualitatively study the cellular biological effects of cell phone radiation, this study simulated cell phone radiation at 1800 MHz, 2 W/kg. All the results are aimed at a dose of 2 W/kg, which is the limit in mobile phone standards adopted by most countries [15]. We believe that EMR first caused a decrease in cell viability, followed by apoptosis due to the accumulation of damage to biomolecules and cell organs. Therefore, EMR within a short period of 36 h may reduce the cell viability, but 48 h of exposure may cause significant apoptosis in NIH/3T3 cells.

In the present study, damage to the mitochondrial structure during apoptosis was found after 12, 24, 36, and 48 h of EMR. Mitochondrial damage included the appearance of swelling and vesicular morphology, which is consistent with previous reports [22]. Mitochondrial fluorescence intensity slightly decreased between the sham and exposure groups after 24 h and 48 h of exposure. Mitochondria are the main cell organs to generate ROS [30] and they play a vital role in cell apoptosis [31]. Previous works have demonstrated that EMR induces apoptosis via the classical mitochondria-dependent caspase-3 pathway [31]. Similar to the results of this study, the mitochondria of rat hippocampi after microwave exposure also showed vesicular and swollen structure [32]; 935 MHz RF-EMF at 4 W/kg for 24 h might lead to impaired mitochondrial function in SH-SY5Y cells [33]. In this study, the number of normal mitochondria (with high mitochondrial membrane potential) may slightly decrease in the exposure group. At the same time, the electron microscopy results of this experiment demonstrate that EMR may also cause mitochondrial damage. Once the mitochondrial structure is damaged, its function may be affected, which might affect physiological and biochemical reactions such as cell energy conversion, cell metabolism, and apoptosis. Therefore, apoptosis caused by EMR may be due to the endogenous apoptotic pathway initiated by mitochondria.

As one of the most important controllers of a cell’s fate, the level of p53 mRNA in the exposure group was 1.4-fold greater than in the sham group after irradiation for 48 h. Though the results of western blot analysis did not show significant differences in p53 protein expression, it is obvious from the immunofluorescence images that p53 fluorescence intensity was enhanced significantly after 24 h and 48 h of exposure. The apoptotic signal of mitochondria often comes from the intracellular transcription factor p53. As a tumor suppressor gene, p53 is kept at a low expression level normally; when the cells encounter stressful situations, p53 is activated and cell growth is arrested by p53 blocking the cell cycle, and even apoptosis is triggered. Consistent with the results of this study, Xing et al. found that 24 h of exposure to microwaves increased the levels of p53 protein [34]; Solek et al. believed that pulsed electromagnetic field induced an increase in the p53 protein level, resulting in p53/p21-dependent apoptosis [35]. However, in contrast to those results, there are negative findings: Hirose et al. demonstrated that 2.1425 GHz EMR caused no significant difference in expression levels of p53 between the exposure group and the control group [36]; Silva et al. indicated that no effect on p53 expression was found [37]. The inconsistency in the data may be due to the different frequencies and times of EMR used in each experiment. The results of this study indicate that 48 h of EMR might upregulate p53 expression in NIH/3T3 cells, which may activate a mitochondria-mediated apoptotic pathway and cause apoptosis.

## 5. Conclusions

In conclusion, this study reveals an increase in the apoptosis rate, upregulation of p53, and mitochondrial damage in NIH/3T3 cells are all caused by 1800 MHz, 2 W/kg EMR. We provide new experimental evidence for the biological effects of EMR on cells and also provide a reference for studying the health effects of long-term EMR exposure.
